# Core Fucosylation Regulates the Function of Pre-BCR, BCR and IgG in Humoral Immunity

**DOI:** 10.3389/fimmu.2022.844427

**Published:** 2022-03-25

**Authors:** Yuhan Sun, Xueying Li, Tiantong Wang, Wenzhe Li

**Affiliations:** ^1^College of Basic Medical Science, Dalian Medical University, Dalian, China; ^2^Division of Regulatory Glycobiology, Institute of Molecular Biomembrane and Glycobiology, Tohoku Pharmaceutical University, Sendai, Japan; ^3^Research Institute for Microbial Diseases and World Premier International Immunology Frontier Research Center, Osaka University, Suita, Japan

**Keywords:** core fucosylation, pre-B cell, BCR, IgG, humoral immune response

## Abstract

Most of the membrane molecules involved in immune response are glycosylated. N-glycans linked to asparagine (Asn) of immune molecules contribute to the protein conformation, surface expression, stability, and antigenicity. Core fucosylation catalyzed by core fucosyltransferase (FUT8) is the most common post-translational modification. Core fucosylation is essential for evoking a proper immune response, which this review aims to communicate. First, FUT8 deficiency suppressed the interaction between μHC and λ5 during pre-BCR assembly is given. Second, we described the effects of core fucosylation in B cell signal transduction *via* BCR. Third, we investigated the role of core fucosylation in the interaction between helper T (T_H_) cells and B cells. Finally, we showed the role of FUT8 on the biological function of IgG. In this review, we discussed recent insights into the sites where core fucosylation is critical for humoral immune responses.

## Introduction

Humoral immune response relies on intrinsic B cell development. The gradual stages of B lymphocyte differentiation are marked by the arrangement and expression of immunoglobulin (Ig) genes in the bone marrow (BM) (1). Early B lymphocytes are produced in the BM, in which there is a stepwise subgroup from hematopoietic stem cells (HSC) to immature B cells. The middle steps are composed of pro-B cells and pre-B cells. The V(D)J rearrangement of the μ-heavy chain (μHC) gene occurs in pro-B cells, and the gene rearrangement of light chains begins in the pre-B cell stage. Once a light chain gene is assembled and a completed IgM is expressed on the surface of immature B cells, the B cells differentiate further to become mature B cells expressing IgD and IgM. The mature B cells recirculate through secondary lymphoid tissues, where they may encounter foreign antigens (Ags). Ag-binding B cells are trapped in the T-cell zone of lymphoid tissue and activated by encounter with helper T (T_H_) cells, and differentiated into plasma cells, which secret large amounts of antibodies (Abs) to provide lasting protective immunity (2, 3).

**Figure 1:**
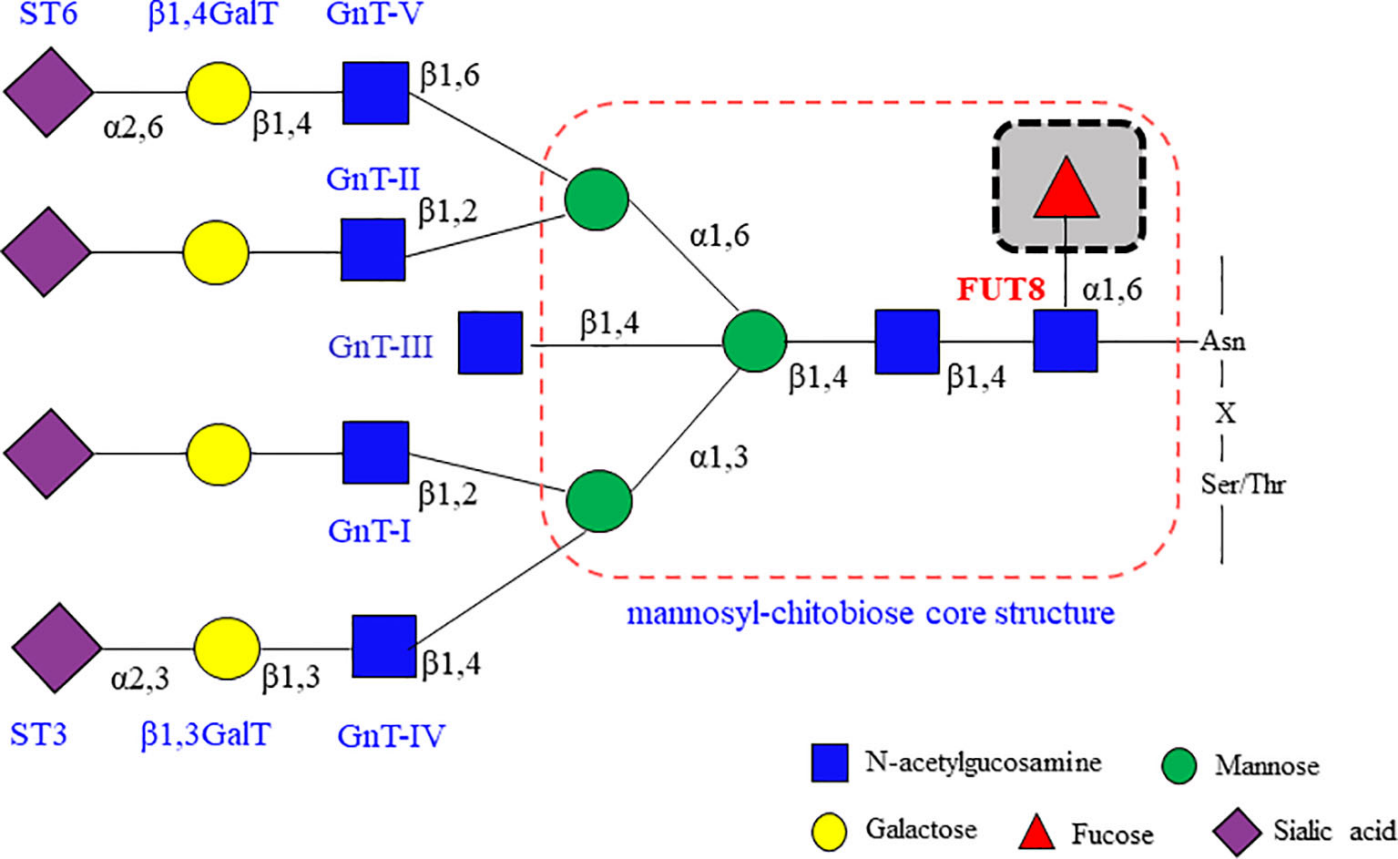
Glycosyltransferases involving in the biosynthesis of branching N-glycans. α2,6 sialyltransferases (ST6) and/or α2,3 sialyltransferases (ST3) that act on galactose, β1,4 galactosyltransferases (β1,4GalT) and/or β1,3 galactosyltransferases (β1,3GalT) that act on N-acetylglucosamine (GlcNAc), and the family of N-acetylglucosamine transferases (GnT-I, GnT-II, GnT-III, GnT-IV, GnT-V and so on) that participate in GlcNAc synthesis. Fucosyltransferases (FUT3, FUT4, FUT5, FUT6, FUT7, FUT8, FUT9) can attach fucose in either α1,3/α1,4 or α1,6 linkage. These enzymes act sequentially, so that the product of one enzyme yields a preferred acceptor substrate for the subsequent action of another. Different proteins may have different subsets of N-glycans, which is referred to as microheterogeneity. All N-glycans share a common core sugar sequence, Manα1–6(Manα1–3) Manβ1–4GlcNAcβ1–4GlcNAcβ1.

Almost all of the immunological molecules are glycosylated (4, 5). Glycosylation is one of the most common post-translational modifications of eukaryotic proteins. The glycans attached to these proteins can be classified into two major types: O-linked glycosylation, whose glycan chains are linked to the oxygen atom of serine or threonine residues in the Golgi, and N-linked glycosylation, whose glycan chains are covalently linked to the amide nitrogen of asparagine (Asn) residues of an Asn-X-Ser/Thr sequence (where X is not proline) by an N-glycosidic bond in the endoplasmic reticulum (ER). Despite their microheterogeneity, most N-glycans share a common N-glycan precursor, 14 monosaccharide residues (i.e., Glc_3_Man_9_GlcNAc_2_) that is pre-assembled on the ER membrane before it is transferred to protein. After the attachment of the N-glycan precursor to Asn-X-Ser/Thr in a protein, a series of processing reactions trim the N-glycan in the ER. After sequential trimming of the terminal monosaccharide residues (glucose and mannose), the glycoproteins exiting the ER en route to the Golgi carry N-glycans with a Man_8_GlcNAc_2_ isomer. Then, the glycoprotein undergoes further sugar chain elongation and “glycan maturation” (e.g., fucosylation, galactosylation, and terminal sialylation) by a suite of glycosyltransferases (6). The glycosyltransferases that assemble monosaccharide moieties of a simple nucleotide sugar donor substrate (e.g., UDP-Gal, GDP-Fuc, or CMP-Sia) into the acceptor substrate (linear and branched glycan chains). The ER-Golgi pathway harbors many glycan-modifying enzymes in eukaryotic cells. In the *medial*-Golgi, the N-glycan has two branches, which are initiated by the addition of two-terminA006C N-acetylglucosamine (GlcNAc) residues by N-acetylglucosaminyltransferase-I (GnT-I) and GnT-II. Additional branches can be initiated at C-4 of the core mannose α1,3 (by GnT-IV) and C-6 of the core mannose α1,6 (by GnT-V) to yield tri- and tetra-antennary N-glycans. N-glycans may carry a “bisecting” GlcNAc residue that is catalyzed by GnT-III. Further sugar additions, such as galactosylation by galactosyltransferases (GalT), fucosylation by fucosyltransferases (FUTs), and sialylation by sialyltransferases (STs), mostly occur in the *trans*-Golgi. FUTs consist of 13 members, including FUT1 to FUT11, protein O-fucosyltransferase 1 (POFUT1), and POFUT2. Core fucosylation is catalyzed by core fucosyltransferase (FUT8). **Figure 1** depicts a bisecting N-GlcNAc on a tetra-antennary N-glycan, which may be present in all of the more highly branched structures. Glycosylation regulates several glycoprotein functions, including half-life on the membrane, sorting to specific subcellular sites, and folding in the ER (7).

We have been devoted to the study that core fucosylation is an essential segment in the early B cell differentiation (8, 9), B cell activation (10, 11), Ab production (11, 12), systemic lupus erythematosus (SLE) development (11), infection (12, 13) and antitumor activity (14). The critical physiological function of the fucosylation is characterized by leukocyte adhesion deficiency II, which emphasizes the physiological significance of the fucosylation due to immunodeficiency caused by fucosylation deficiency (15). This review focuses on the unique roles of core fucosylation in adaptive humoral immune responses.

## Core Fucosylation and Its Function

**Figure 2:**
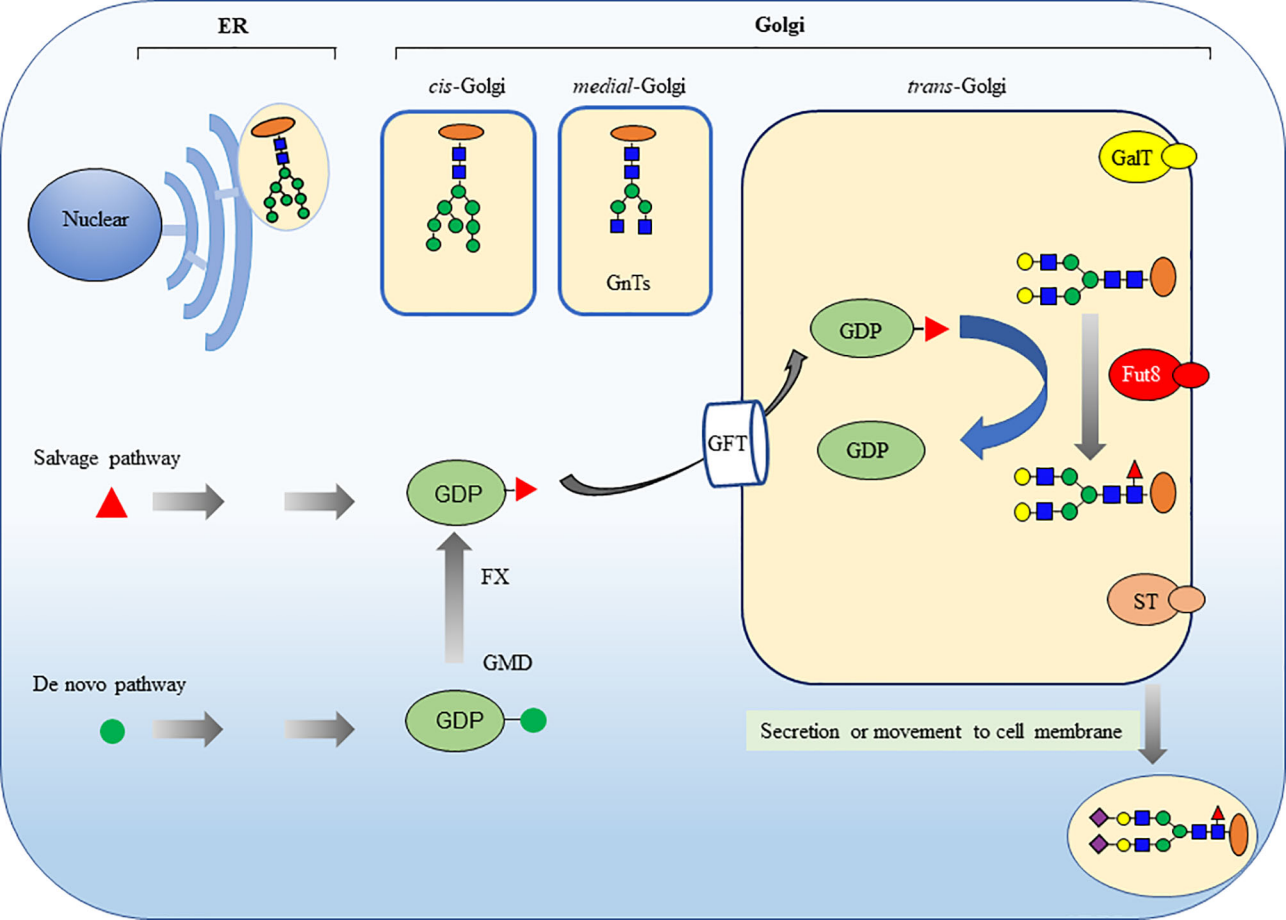
Core fucosylation modification in mammalian N-glycans is catalyzed by FUT8. The ER-Golgi pathway harbors many glycan-modifying enzymes in eukaryotic cells. The glycosyltransferases that operate in the ER are woven into the ER membrane. In contrast, the glycosyltransferases in Golgi compartments are generally type II membrane proteins with a small cytoplasmic amino-terminal domain, a single transmembrane domain, and a large lumenal domain that has an elongated stem region and a globular catalytic domain. FUT8 is a typical type II membrane protein and a Golgi apparatus–resident glycosyltransferase to catalyze the transfer of a fucose from GDP-fucose to the inner GlcNAc of N-glycans through α1,6-linkage (core fucosylation). There are two different pathways for producing GDP-fucose, namely salvage pathway and *de novo* pathway. The GDP-fucose is transferred from the cytosol into the Golgi apparatus by GDP-fucose transporter (GFT). The sugar additions, such as galactosylation by GalT, fucosylation by FUTs and sialylation by STs, mostly occurring in the *trans*-Golgi.

In mammalian cells, the core fucosylation catalyzed by FUT8 is a common post-translational modification (**Figure 2**) (16, 17). FUT8 is the only glycosyltransferase that catalyzes the transfer of fucose from GDP-fucose to the inner GlcNAc of N-glycans through α1,6-linkage (core fucosylation) in the Golgi apparatus. There are two different pathways for producing GDP-fucose, namely the salvage and *de novo* pathways. The salvage pathway utilizes free fucose derived from dietary sources. The *de novo* pathway (the major way) synthesizes GDP-fucose from GDP-mannose through oxidation, epimerization, and reduction catalyzed by two enzymes, including GDP-mannose 4,6-dehydratase (GMD) and GDP-4-keto-6-deoxy-mannose-3,5-epimerase-4-reductase (FX). Then, the GDP-fucose transporter (GFT) transfers the GDP-fucose from the cytosol into the Golgi apparatus.

Previous studies have observed relationships between protein structures and the N-glycan repertoires (18). The underlying pentasaccharide (mannosyl-chitobiose: Man3GlcNAc2) core structure of all eukaryotic N-glycans serves as scaffolding. The flexible glycosidic linkages are often found in the pentasaccharide core structure of the N-glycans. The antenna flexibility could play a specific role in directing precise recognition in carbohydrate-protein interactions. N-linked glycosylation does not induce any secondary structure of proteins but that it does alter their conformational preferences in the vicinity of the glycosylation site, increasing the probability of more compact conformations (19). These effects seem to involve only the monosaccharide units of the N-glycan and are probably mediated by steric and hydrophobic or hydrophilic interactions between the N-glycan and the neighboring amino acid sidechains (20). Most glycoproteins are expressed on the cell surface. Each interaction between such proteins involved in cell-cell recognition events are frequently weak. Strong cell-cell interactions are obtained from many of these interactions occurring simultaneously on cell surface molecules. The N-glycans on these molecules influence their orientation and their packing of glycoproteins on the cell surface.

It has been reported that the core fucose influences the conformational flexibility of the core-fucosylated biantenna of N-glycans (21). The core fucose also plays a crucial role in the binding of an N-glycan to a plant lectin (22). As shown in the **Table 1**, core fucosylation regulates the conformation, stability and expression of immune molecules, such as pre-BCR, BCR, IgG, T cell receptor (TCR), CD276(B7H3), CD14, CD41a, hemagglutinin (HA), PD-1 and VLA-4. The association between an N-glycan and its Asn sidechain is relatively rigid and planar, with a tendency to extend the core sugar structure (24). Conversely, core fucosylation is strongly influenced by the structure of the proteins. The glycosylation sites of the proteins and their subcellular location determine whether the conjugated N-glycans will be modified by core fucosylation. It is reasonable to propose that core fucosylation could affect the glycosidic linkage flexibility and conformation of proteins, resulting in modification of protein interactions or assembly. Core fucosylation is ubiquitously expressed in mammalian tissues and participate in the regulation of numerous biological events of physiological and pathological conditions (25), including cell growth (8, 26), cell signal transduction (9, 10), protein-protein interaction (9, 10), cell-cell interaction (11, 12) and tumorigenesis (23, 27, 28). FUT8 knockout (FUT8^-/-^) mice exhibit early postnatal death (29), retardant growth (26, 30), emphysema-like changes (29, 31), schizophrenia-like phenotype (32) and so on.

**Table 1:** Core fucosylated immune molecules and their crucial functions.

Immune molecules	Functions	References
pre-BCR	Loss of core fucosylation of μHC reduced the binding affinity between μHC and λ5.	(13, 14)
BCR	Loss of core fucosylation reduced the lipid raft formation of B cells.	(15)
IgG	Loss of core fucosylation promotes the ADCC of nature killer cells.	(6–8)
TCR	Core fucosylation of the TCR is necessary for T-cell of nature killer cells.	(3–5)
CD276(B7H3)	FUT8-B7H3 axis is a promising strategy for improving anti-tumor immune responses.	(9)
CD14	Core fucose is critical for CD14-dependent Toll-like receptor 4 signaling.	(1)
CD41a	The enzyme activity of FUT8 is involved in the megakaryocytes differentiation.	(16)
Hemagglutinin (HA)	Loss of core fucosylation reduces the binding of influenza A virus HA to IgE.	(12)
PD-1	Loss of core fucosylation enhanced the PD-1 expression in CD8^+^ cytotoxic T lymphocytes.	(23)
VLA-4	Loss of core fucosylation decreased binding between pre-B cells and stromal cells.	(13)

## Core Fucosylation Regulates Pre-BCR Assembly

During early B cell differentiation, the formation of pre-BCR complex allows progression from the pro-B cell stage to pre-B cell stage (33). The assembly of the pre-BCR on the cell membrane is a critical checkpoint for B cell growth and differentiation in humans and mice (34–38). Suppression of pre-BCR complex formation inhibits the transition of B lymphocyte development from pro-B cell stage to pre-B cell stage (39, 40). The functional pre-BCR complex consists of immunoglobulin (Ig) μHC, surrogate light chain (SLC), and Igα/Igβ (CD79a/CD79b) (41). The μHCs assembled with SLC are classically obligatory for pre-BCR trafficking to the surface (42). The μHC (GI:90956) comprises the variable region of Ig μHC (V_H_) and the constant portion of the Ig μHC (C_H_). The λ5 (GI:54887631) and Vpre-B are non-covalently associated to form SLC. In a pre-BCR complex, λ5 binds to the C_H1_ domain of μHC, while Vpre-B interacted with the V_H_ domain. Vpre-B is stabilized by a salt bridge between Glu^106^ residue of Vpre-B and Arg^59^ residue of V_H_ (33). The N-glycans on μHC are of the high-mannose type (43). Ubelhart et al. (44) also discovered that μHC of mouse and human Igs contains the N-linked glycosylation site N^46^ and that a conserved N^46^-glycan at the C_H1_ domain of μHC was necessary for the interaction of μHC and λ5, followed by the formation of pre-BCR. There are no N-glycosylated sites on λ5, and it is covalently coupled to the C_H1_ domain of μHC *via* a carboxyl-terminal cysteine (42). Only pre-B cells that express the pre-BCR complex undergo the following clonal expansion.

**Figure 3:**
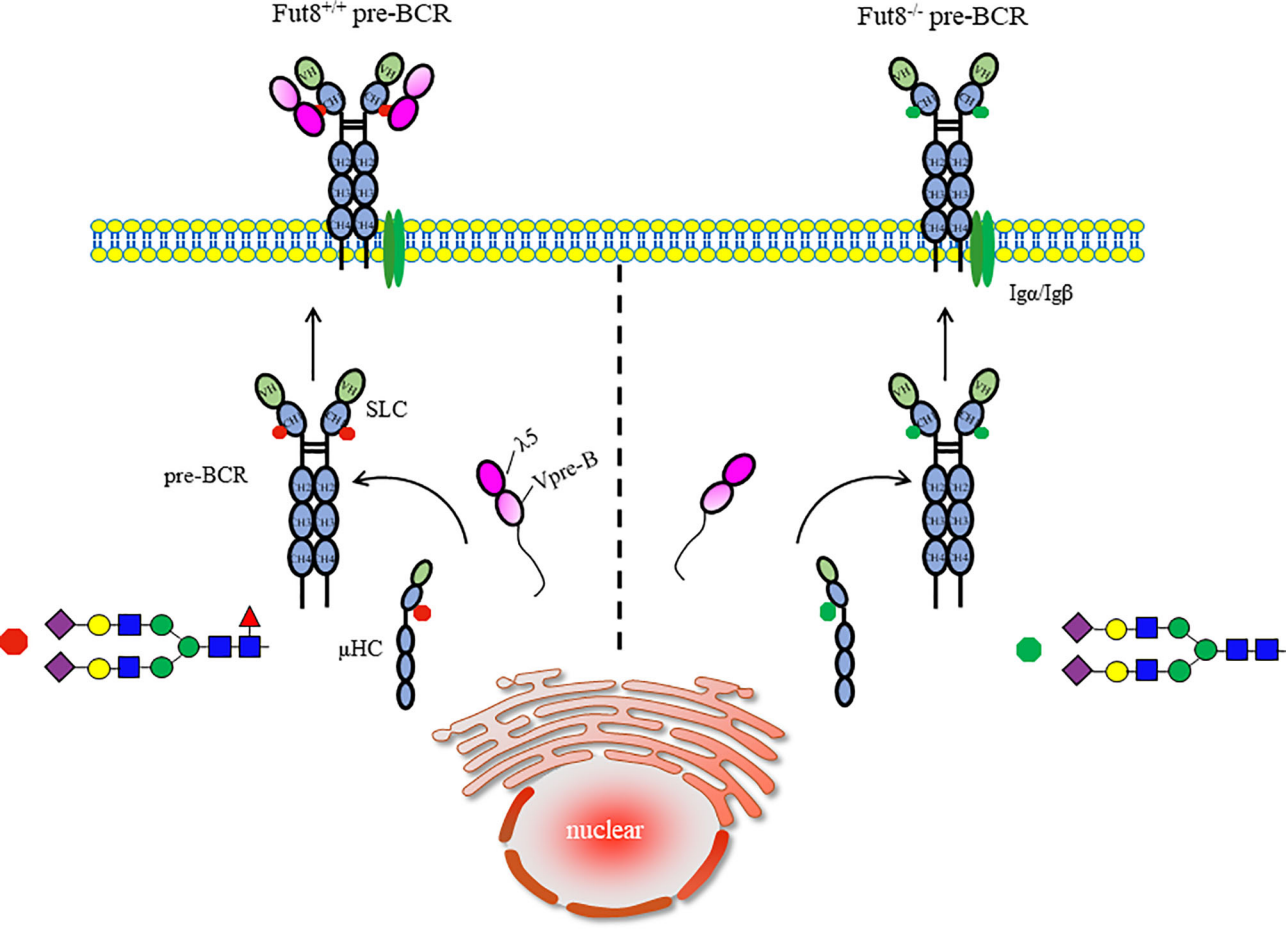
Core fucosylation regulates the assembly of pre-BCR. The early B cells growth depends on the assembly of pre-BCR. The conserved N^46^-glycosylation site on C_H1_ domain of μHC played a critical role in modulation of the interaction of μHC and λ5 before the formation of pre-BCR. Removal of core fucose of μHC impaired the interaction between μHC and λ5, and pre-BCR assembly, which is necessary and sufficient for signal transduction *via* pre-BCR and early B cell proliferation.

In FUT8^-/-^ BM, we found a profound and selective suppression in pre-B cell generations without a concomitant change in the pro-B cell population (8). To address the impairment of the pre-B cell population in FUT8^-/-^ mice, we investigated the role of FUT8 in pre-BCR assembly. Indeed, μHC is a core fucosylated protein, and loss of core fucosylation of μHC reduces the binding affinity between μHC and λ5. Consistent with this result, the formation of pre-BCR is down-regulated in the FUT8 knockdown pre-B cell line (70Z/3-KD cells) and has been recovered in 70Z/3-KD-re cells (9). Indeed, the subpopulation of μHC^+^λ5^+^ cells is lower in FUT8^-/-^ BM than in FUT8^+/+^ BM. Early pre-B cells expressing both μHC and SLC are in the BM and fetal liver; one produces SLC-pairing μHC, and the other produces SLC-nonpairing μHC (36). Only half of the in-frame rearranges μHCs pair correctly with SLCs (45). Subsequently, μHCs that bind poorly to SLCs are subsequently poorly represented among μHC families (46). The μHC undergoes glycosylation modification and is transported to the cell surface. The newly synthesized membrane form of μHC is Endo H-resistant, which reaches the cell surface, while the secretory form is Endo H-sensitive (47). Since FUT8 expression is significantly increased during the transition from pro-B to pre-B cell development (8), and since the core fucosylation is required for pre-BCR complex assembly (9), it is conceivable that FUT8 controls the core fucosylation of μHC, followed by pre-BCR complex assembly. There are no N-glycosylation sites in λ5, Vpre-B, and V_H_ domains of μHC. When the binding of V_H_ of μHC to Vpre-B is a protein-protein interaction by ionic bonding between Glu^106^ of Vpre-B and Arg^59^ residue of V_H_ region, and the binding of C_H1_ of μHC to λ5 is a protein-glycoprotein interaction. It is reasonable to propose that the FUT8 regulated the interaction between C_H1_ of μHC and λ5, but did not influence the interaction between Vpre-B and μHC during pre-BCR assembly (**Figure 3**). The pre-BCR tunes pre-B cell repertoire by driving the preferential differentiation and expansion of cells with a higher quality of μHC (37). Thus, we propose a mechanism in which core fucosylated μHC has a higher potential to assemble with SLC and amplify the pre-B cell repertoire.

Pre-BCR expression is sufficient and required for constitutive cell signaling (48). Mutations of genes associated with the pre-BCR signal pathway culminate in agammaglobulinemia (35–38, 49). Core fucosylation is required for the appropriate binding of μHC to λ5, as well as pre-BCR assembly (9). Except for the signal transduction *via* pre-BCR, integrins bind to vascular adhesion molecule-1 (VCAM-1), thereby facilitating signaling with stimulation of IL-7 from stromal cells, which plays a significant role in the generation of B cell precursors (50). Integrins on pre-B cells, together with pre-BCR and galectin-1, could form a homogeneous lattice at the contact area between pre-B cells and stromal cells (51). FUT8 ablation reduces binding affinity between very late antigen 4 (VLA-4, α4β1 integrin) on pre-B cells and VCAM-1 on stromal cells, which impaired the colony expansion of pre-B cells in FUT8^-/-^ BM (8).

## Core Fucosylation Regulates the Signal Transduction *via* BCR

Upon B cell activation, B cells recognize both soluble and membrane-associated Ags by B-cell receptor (BCR), and respond to them (3). After the recognition of Ags, BCRs on B cells trigger signaling that eventually induced B cell activation and Abs production (52). First identified in 1970 (53), the BCR and Ab consisted of two heavy (H) chains and two light (L) chains, which are linked by disulfide bonds. Each H chain is composed of an N-terminal variable domain (V_H_) and three constant domains (C_H1_, C_H2_, C_H3_); the L chains consist of an N-terminal variable domain (V_L_) and one constant domain (C_L_). The ‘‘fragment antigen-binding’’ (Fab) domains, which mediates antigen recognition, contain the complementarity determining regions (CDRs), located in the N-terminal region of C_H_ and C_L_, which define the antigen-specificity. The “fragment crystallizable’’ (Fc) of the C-terminal regions is composed of the two C_H_ (C_H2_, C_H3_), which bind to the immune effector molecules, including Fc receptors. The BCR complex contains a membrane-bound immunoglobulin (mIg) with a small cytoplasmic domain and a disulfide-linked heterodimer Igα–Igβ (CD79a-CD79b), which contains the immunoreceptor tyrosine-based activation motif, ITAMs (54, 55).

There are glycosylation sites at Asn^297^ of the Fc region but not in the V_H_ of IgG-BCR. Microheterogeneity of N-glycans on IgG is detected by the linkage of galactose–sialic acid or galactose at one or both of the terminal GlcNAc or linkage of a third GlcNAc arm. The profile of N-glycans at Asn^297^ on BCR is of the complex type (51). Furthermore, BCR is a highly core fucosylated glycoprotein, and the core fucosylation regulates the function of BCR to distinguish Ag (10). Indeed, FUT8 ablation impaired the binding affinity of IgG-BCRs to their ligands. Since core fucosylation could modulate the conformation and ligand affinity of the membrane receptors (8–11, 26, 29) and could influence the flexibility of N-glycans antenna (21), it is suggested that core fucosylation could control the axial rotation of the Fab arms and the recognition of BCR to Ag. Core fucosylation of μHC impaired the interaction between μHC and λ5 of the SLC. However, FUT8 gene knockout/knockdown does not influence the BCR expression on the cell surface. It is conceivable that the difference of N-glycan in heavy chains is attributed to the distinct assembly between pre-BCR and BCR.

**Figure 4:**
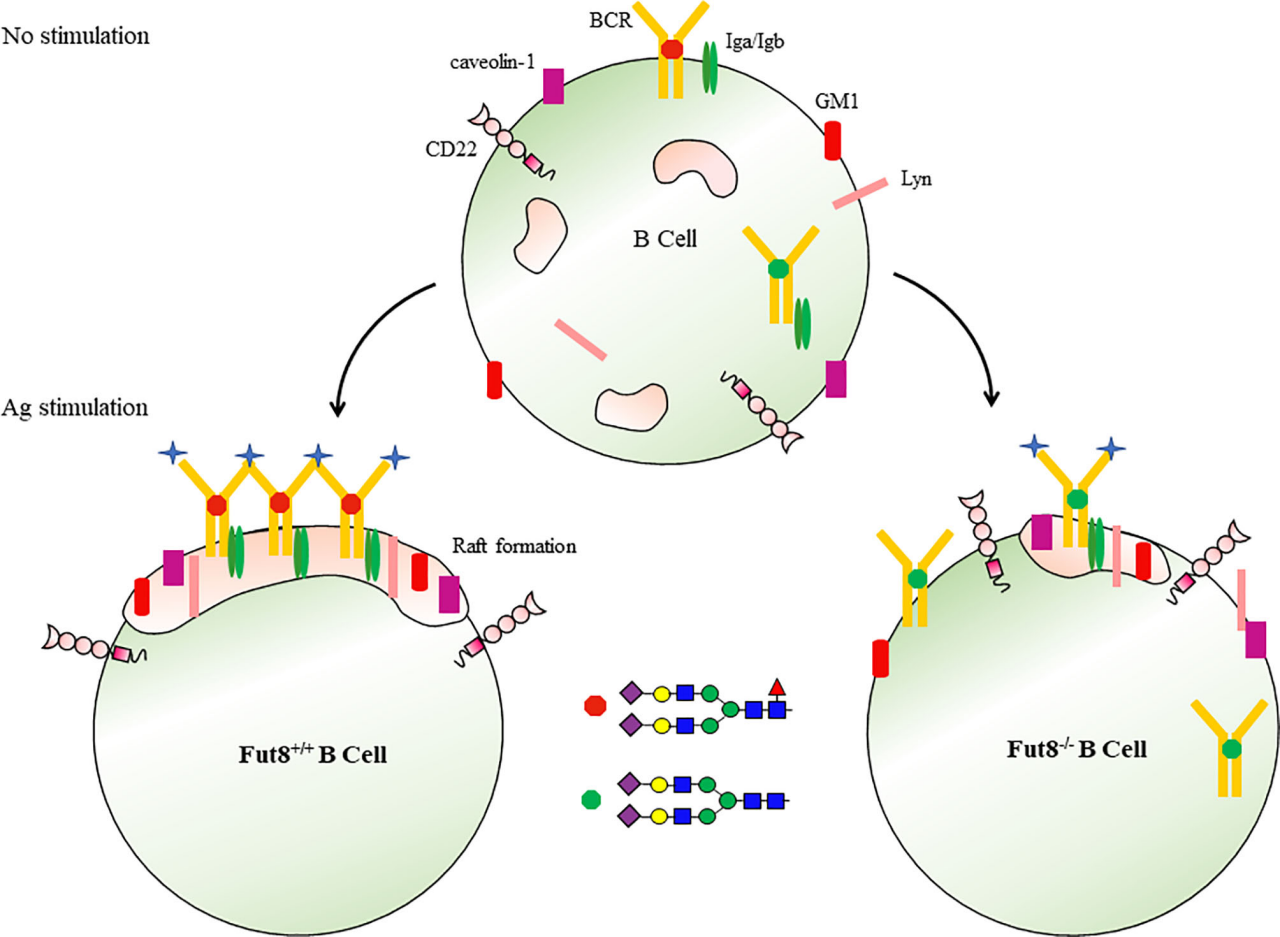
Core fucosylation regulates the signal transduction *via* BCR. Upon Ag engagement, BCRs on the surface of B cells associated with lipid rafts and triggers signal transductions required for the activation of B cells. Core fucosylation promoted B cells to form a highly efficient lipid raft domain, thereby regulating signal transduction *via* BCR.

Lipid rafts are heterogeneous, dynamic and transient plasma membrane entities enriched in saturated phosho-, sphingo- and glycolipids, cholesterol, lipidated proteins and glycosylphosphatidylinositol (GPI)-anchored proteins (56). The BCR is found in detergent-soluble membrane fractions in resting cells, but is acquired in detergent-resistant membrane fractions (raft domains) after receptor activation (57–59). Fluorescence resonance energy transfer confocal microscopy revealed that the association of BCR with lipid raft occurred within seconds, resulting in the cross-linking of BCR (60). Upon Ag stimulation, core fucosylation influences the binding of BCR to Ag peptide at 30 seconds, the formation of lipid raft at 3 min, and signal transduction *via* BCR after 5 min (10). FUT8 ablation suppresses the together with BCR, Lyn, and GM1 or caveolin-1 in lipid raft domains. Except for their role in signaling molecules concentrating, lipid rafts play a significant role in protein internalization through the endocytic pathway (60). The FUT8 gene disruption significantly reduces the Ag endocytosis of B cells (10). Several mechanisms may contribute to the reduced lipid raft formation by FUT8 ablation. First, because of the conformation with an increasing affinity for lipid rafts caused by Ag-driven oligomerization of the BCR (58), the failed oligomerizations of BCR by afucosylation result in reduced lipid raft formation. Second, caveolin-1 is a scaffolding protein of cholesterol-rich caveolae lipid rafts (57), the inhibited recruitment of caveolin-1 by FUT8 ablation influences the efficient coalescing of the lipid raft in the plasma membrane. Third, the depletion of core fucose increased the hydrophilicity of immune molecules and impaired lipid raft formation (61) (**Figure 4**). Core fucosylation controls several parameters associated with B cell activation, including Ag recognition, BCR oligomerization, signal transduction *via* BCR, and lipid raft cluster formation.

## Core Fucosylation Regulates the T_H_-B Cell Interaction

Upon Ag engagement by BCR, B cells process and present the Ag peptide in association with the major histocompatibility complex class II (MHC-II) molecules *via* BCR-mediated endocytosis (3). Then, the peptide-loaded MHC-II complex (pMHC-II) on B cells can be recognized by Ag-specific armed T_H_ cells *via* T cell receptor (TCR) (62). During T_H_–B cell interaction, activated T_H_ cells produce cytokines for the B lymphocytes clonal expansion and differentiate into Ab-secreting cells (58). Several studies revealed the contribution of glycosylation in the T_H_-B cell interaction (20). Loss of GlcNAcT-V glycosyltransferase (GnT-V), for example, could promote the recruitment of TCR to the synapse and enhance TCR internalization (63). FUT1 overexpressed T cells enhance the signaling pathway *via* TCR and apoptosis required for thymocyte maturation arrest (64). The α2,6-sialyltransferase (ST6Gal-1) is required for the development of humoral immunity (65, 66). The removal of N-glycan in T cells improves the functional avidity of TCR to Ag recognition (67). Notably, the most immune molecules participated in T and B cell activation are core-fucosylated glycoproteins. To assess the function of FUT8 in the T–B cell interactions, we obtained FUT8^+/+^OT-II and FUT8^-/-^OT-II mice by crossing FUT8^+/-^ mice with OT-II mice (these transgenic mice express CD4^+^ TCR specific for chicken ovalbumin 323-339, OVA_323–339_). Most key proteins associated with Ag recognition and the orchestration including MHC-I and MHC-II, are glycosylated (68). Although I-A^d^ has a single N-glycan (69), core fucosylation cannot change the OVA_323–339_ presentation abilities of MHC-II (11, 12). However, compared to FUT8^+/+^OT-II cells, the communication of T–B cells (MHC-II^+^ TCRβ^+^ cells) is significantly decreased in FUT8^-/-^OT-II cells (11). Also, ZAP-70 and Syk phosphorylation was significantly reduced in FUT8^-/-^OT-II cells with interaction by OVA_323–339_-loaded B cells. Given that the pMHC on B cells induces a particular conformational change of TCR on T cells (70, 71), and the core fucose is expressed on the cell surface of T and B cells and could affect the flexibility and the conformation of proteins (21). It is reasonable to propose that core fucosylation would influence the conformational stability and geometry of any TCR–pMHC clusters in the T_H_–B cell interactions. Alternatively, a diverse range of glycan-binding proteins, such as galectins and siglecs, bind to the sugar chains on these glycoproteins, thereby modulating cell signaling and cell-cell interactions (21). Galectin-1^-/-^ mice show abnormal thymocyte selection, causing alteration in T cell responses (72). CD22 selectin, which recognizes α2,6-linked sialylated glycans, is a B cell co-receptor that decreases the signaling *via* BCR (73). Fucose-specific lectins (74) enable to participate the events in the interactions between T and B cells.

Ab production is one of the major events in the adaptive humoral immune response. The titer of anti-OVA IgG is markedly decreased after the OVA immunization in FUT8^-/-^ mice (11). Moreover, compared to FUT8^+/+^ SPLs, the number of IgG-producing cells is obviously decreased in FUT8^-/-^ SPLs. During *S. typhimurium* infection, the production of IgG and sIgA specific for bacteria is also decreased in FUT8^-/-^ mice with attenuated the T_H_–B cell interactions, but not in communication between T cells and DCs (12), indicating that core fucosylation is essential for the efficient Ab production. Ig class-switching recombination generates an isotype-switched Abs, which are essential for mediating effective humoral immunity (75). The isotype switching of a mature B cell *via* its BCR from one class to another depends upon the interaction of Ag-stimulated B cells with T_H_ cells. To assess FUT8 function in Ig class-switching, IgGs of different subclasses (class-switched) and IgM (non-switched) are measured in the sera of FUT8^-/-^ mice. The amounts of IgG subclasses, IgG_1_, IgG_2a_, IgG_2b_, and IgG_3_ are significantly reduced in FUT8^-/-^ mice due to low levels of cytokines, such as IL-4, IL-5, IL-6, IFNγ, and TGF secreted by FUT8^-/-^T_H_ cells, while amounts of IgM are relatively normal (11). Core fucosylation plays a crucial role in all steps required for Abs production. First, in the T_H_-B cell interactions, core fucosylation regulates the geometry and conformation of TCR-pMHC clusters in lipid rafts. Second, in cytokine production, core fucosylation controls the expression level of T_H2_-type cytokines (IL-4, IL-6) associated with the activation of T_H_ and B cells (12). Third, in B cell generation, FUT8 affects the population of CD19^+^ B cells.

Several studies have been reported that aberrant cues of glycosylation contribute to autoimmune disease (AD) pathogenesis (76–78). For example, the low levels of sialylated IgG are detected in the sera of rheumatoid arthritis and Wegener’s granulomatosis patients, while the sialylated IgG is increased during remission (78, 79). Galactosylated IgG1 is significantly reduced in the sera of rheumatoid arthritis patients (77). GnT-V ablation induces the coclustering of TCRs at the cell surface, reducing the threshold for T cell activation and causing multiple sclerosis (63). SLE is a typical autoimmune disease whose characteristics are inflammatory disorders and autoantibodies production (80). The level of core fucosylated IgG is significantly increased in the sera of SLE patients (11, 76, 81). Indeed, T_H_ cell activation in the peripheral blood of SLE patients plays a crucial role in SLE pathogenesis, and the hyperactivity of B cells is T_H_ cell-dependent in SLE (82, 83). The remarkable differences of glycosylation on T_H_ cells have been observed in active SLE patients (84). Core fucosylation is required for TCR activation in T_H_ cells (61). Increased-core fucosylation induces T_H_ cell hyper-activation and contributes to the SLE severity and pathogenesis.

## Core Fucosylation Regulates the Function of IgGs

Glycosylation occurs normally in human IgGs, and plays a significant role in IgG function (85–87). Structural evidence proved that each IgG molecule has a highly conserved N-glycan at Asn^297^ in the C_H2_/C_H3_ domain of Fc regions, and plays a crucial role in sustaining the conformation of Fc domain of IgGs. Multiple sugar chain moieties extend from Fc domains toward each other into the interchain region of IgG, and then they stabilize the IgG framework (88). Differences in the sugar chains determine the variances in the orientation of the protein surface (88).

**Figure 5:**
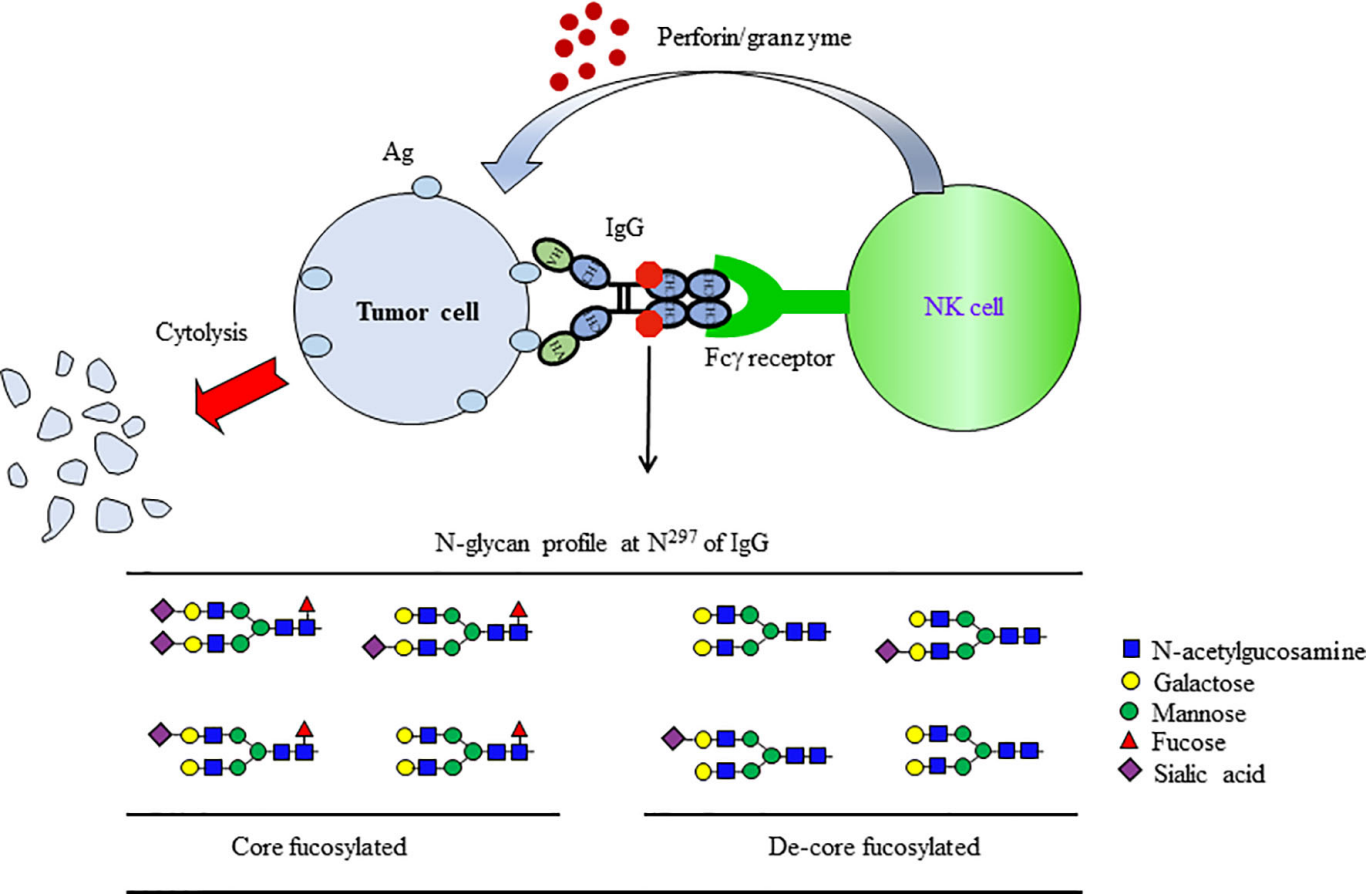
De-fucosylated IgG-Fc domain enhanced the induction of ADCC. ADCC is a specific effector mechanism of natural killer (NK) cells. ADCC refers to the killing of a target cell, which is coated with Abs by NK cells of the immune system. When the antibodies bind to the target Ag (tumor Ag), the IgG Fc is recognized by NK cells *via* FcγRs on their surfaces. Following recognition, the NK cells release chemicals, which then lyse and kill the target cell bound to the Ab. The removal of core fucosylation at Asn^297^ on IgG_1_ results in a 50~100-fold enhancement of ADCC.

Several functions of IgGs are mediated through Fcγ receptors (FcγRs) on the effector cells. Because the N-glycan at Asn^297^ on the Fc domain is located next to the FcγRs binding interface, glycosylation controls the biological function of IgGs (88). The Fc domain sialylation triggers conformational changes in IgG1 that enable interactions with type II FcγRs, while core fucose alters type I FcγRs binding of IgG1 by modulating the Fc’s affinity for FcγRIIIa. Core fucosylated N-glycans attached to the Fc region is a critical determinant of antibody-dependent cell-mediated cytotoxicity (ADCC), as the deletion of core fucose from the Fc region enhances its binding affinity to the FcγRs and significantly improves ADCC (**Figure 5**) (89–92). The highly de-fucosylated (∼60%) IgG_1_ exhibits 100-fold ADCC compared to hyper fucosylated (∼10% defucosylated) IgG_1_, without any difference for Ag binding. Dengue virus infection increases the afucosylation level of IgG_1_, and the level of afucosylated IgG_1_ could predict the severity of dengue disease (93). Moreover, afucosylated IgG_1_ plays a critical role in immune responses to enveloped viruses, including COVID-19 (94). Also, afucosylated IgG efficiently induced FcγRs-dependent natural killer (NK) cell degranulation in malaria (95). As a result of these advances, Ab “glycoengineering” is currently gaining attention as an approach to enhance the effects of therapeutic Abs for tumor and virus infection (96).

In human serum, FUT8 is derived in about 95% from blood platelets (97). Platelets release the FUT8 with components of vesicles during blood coagulation (98). Serum IgG contains more than 30 different N-glycan profiles, and all of the major N-glycans were core fucosylated (99). Core fucosylated glycans is increased on the IgG of SLE patients (81). The hyper-core fucosylation frequently occurs in the sera of epithelial ovarian cancer patients with cisplatin resistance (23) and lung adenocarcinoma patients (14), but the core fucosylation level is down-regulated in the sera of patients with cervical cancer (100). Core fucosylation is also increased in intestinal tissues of patients with inflammatory bowel disease (61).

## Conclusion and Perspectives

Appropriate B cell responses are critical for adaptive humoral immunity. Early B cell differentiation, activation and population of B cells, T_H_-B cell interaction and Ab production are processes carefully orchestrated by a complex network of immune molecules. During B cell development, approximately 90% of B cells are eliminated due to the greatly restricting mature immune repertoire of BCR available for Ag recognition (101). Hence, the mechanisms regulating B cell development are crucial in treating of ADs and improving vaccination strategies.

FUT8 can modify multiple proteins, and core fucosylation of N-glycans of the immune molecules could significantly alter their functions (14). Based on our previous study, core fucosylation is not only associated with pre-BCR assembly and Ag recognition of BCR but also plays a crucial role in effective lipid raft formation, T_H_–B cell interaction, and Ab production in guiding B lymphopoiesis to shape humoral immunity. It is worth noting that the loss of FUT8 inhibits effective Ab production, while hyper-activation of these cells results in SLE pathogenesis. There are 2937 single-nucleotide polymorphisms (SNPs) in the FUT8 gene region, and the FUT8 gene rs35949016 SNP could affect FUT8 expression (51, 102). Given that the balance between specific and degenerate immune responses holds a vital illumination for protective immunity versus autoimmunity, the relevance of FUT8 SNPs with immune regulatory activity will be considered. Several core fucosylated immune molecules have been identified thus far, and their significance in immune responses is becoming clear. However, the following points seem to be limitations of the research on core fucosylation. First, because FUT8 could regulate multiple glycoproteins, the widespread changes in core fucosylated proteins in FUT8^-/-^ cells make it difficult to discern the role of core fucosylation in individual core fucosylated proteins. Second, very few core fucosylated glycoproteins crystallize for the entire N-glycan due to glycan microheterogeneity and flexibility. Third, due to the potential of flexibility in glycosidic linkages, the conformational requirements for efficient binding of core fucosylated N-glycans to proteins are difficult to analyze. The future ability to determine the sugar chains of immune receptors combined with the ability to selectively modulate cellular glycomes is expected to offer exciting chances to regulate adaptive immune responses. With a better understanding of how FUT8 regulates the adaptive immune system, its use in therapy for autoimmunity proves to be a helpful intervention from glycoimmunological aspects.

## Author Contributions

YS and XL were mainly responsible to write the review and complete the Figures. TW collected and checked the References. WL designed the research and modified the manuscript. All authors reviewed and approved the final version of the manuscript.

## Funding

WL is supported by grants from the National Nature Science Foundation of China (32171279, 31870797, 31570797, 31270864, 30972675) and Liaoning Provincial Program for Top Discipline of Basic Medical Sciences.

## Conflict of Interest

The authors declare that the research was conducted in the absence of any commercial or financial relationships that could be construed as a potential conflict of interest.

## Publisher’s Note

All claims expressed in this article are solely those of the authors and do not necessarily represent those of their affiliated organizations, or those of the publisher, the editors and the reviewers. Any product that may be evaluated in this article, or claim that may be made by its manufacturer, is not guaranteed or endorsed by the publisher.
